# Two Independent Contributions to Step Variability during Over-Ground Human Walking

**DOI:** 10.1371/journal.pone.0073597

**Published:** 2013-08-28

**Authors:** Steven H. Collins, Arthur D. Kuo

**Affiliations:** 1 Department of Mechanical Engineering and Robotics Institute, Carnegie Mellon University, Pittsburgh, Pennsylvania, United States of America; 2 Departments of Mechanical Engineering and Biomechanical Engineering, University of Michigan, Ann Arbor, Michigan, United States of America; University of California, Merced, United States of America

## Abstract

Human walking exhibits small variations in both step length and step width, some of which may be related to active balance control. Lateral balance is thought to require integrative sensorimotor control through adjustment of step width rather than length, contributing to greater variability in step width. Here we propose that step length variations are largely explained by the typical human preference for step length to increase with walking speed, which itself normally exhibits some slow and spontaneous fluctuation. In contrast, step width variations should have little relation to speed if they are produced more for lateral balance. As a test, we examined hundreds of overground walking steps by healthy young adults (*N* = 14, age < 40 yrs.). We found that slow fluctuations in self-selected walking speed (2.3% coefficient of variation) could explain most of the variance in step length (59%, *P* < 0.01). The residual variability not explained by speed was small (1.5% coefficient of variation), suggesting that step length is actually quite precise if not for the slow speed fluctuations. Step width varied over faster time scales and was independent of speed fluctuations, with variance 4.3 times greater than that for step length (*P* < 0.01) after accounting for the speed effect. That difference was further magnified by walking with eyes closed, which appears detrimental to control of lateral balance. Humans appear to modulate fore-aft foot placement in precise accordance with slow fluctuations in walking speed, whereas the variability of lateral foot placement appears more closely related to balance. Step variability is separable in both direction and time scale into balance- and speed-related components. The separation of factors not related to balance may reveal which aspects of walking are most critical for the nervous system to control.

## Introduction

During what might appear to be steady human walking, each step actually varies slightly in timing, length, width, and other measures. This step variability may be caused by a number of factors related to the physical body and the nervous system, making its examination potentially valuable not only for understanding how locomotion is produced and controlled, but also for indicating functional walking ability such as the risk of falls in elderly or impaired individuals [1,2]. One limitation is that an observation of variability might plausibly be attributed to factors as diverse as balance control [3], neural rhythmicity [4], or even psychological factors such a confidence [1,5] and cognitive demand [6], to name a few. If a direct dependency between a causative factor and its effect could be established, it might be possible to decompose gait into separable components, which might then help functionally characterize walking ability. Here we examine how balance and the regulation of overall walking speed may contribute independently to step variability in normal human gait.

Balance control may contribute to step variability if foot placement is adjusted step-by-step to induce stabilizing corrections to body motion [7,8]. We have previously proposed that step adjustments, particularly in the lateral (side-to-side) direction, are performed actively by the central nervous system (CNS) based on integrative sensory feedback (from visual, vestibular, proprioceptive, and other sensors [9]), because the dynamics of walking appear to be quite unstable in that direction [3,10]. In contrast, fore-aft step variations might be driven more by the pendulum-like dynamics of the legs with local reflex control [10], which may be sufficient to dynamically stabilize gait in the sagittal plane [11] but not laterally [12]. The difference between integrative and local control suggests that there should be a higher sensitivity of lateral foot placement to reduced or perturbed visual feedback than for fore-aft foot placement. Indeed, studies of both over-ground and treadmill walking show that the fore-aft component of step variability is smaller than and less dependent on visual feedback than the lateral component [3,10,13,14]. Both directions of step adjustments appear to contribute to balance on the relatively fast time scale of single steps, but in different ways.

Step variability may, however, have other contributions less related to balance. In particular, one possible cause for step variability is spontaneous variation in overall walking speed. Humans can select different walking speeds, and may at times be observed to walk at non-uniform speed [15], perhaps influenced by factors as disparate as conscious task goals, the distraction of attention by secondary tasks [6], and the subconscious tendency to minimize energy expenditure [16]. Whatever their cause, walking speed fluctuations may cause steps to vary, simply because humans prefer a particular step length for a given speed. In fact, the preferred combination of step length and frequency coincides with the minimum energy expenditure for a given speed [16–18]. Over a step or two, balance-related adjustments to foot placement might be expected to override this relationship, making instantaneous speed somewhat dependent on step-by-step foot placement. But over the time scale of many steps, slow fluctuations in overall walking speed would be expected to contribute substantially to step variability. A number of studies have identified long-term fluctuations in parameters such as stride rate that cannot be explained by random white noise and therefore appear to be partially deterministic [19–21]. These fluctuations occur over time scales of ten strides or more [22], making them unlikely to contribute to step-by-step balance. Other measurements show that walking speed, step length, and step frequency also exhibit long-range correlations [23]. Perhaps speed fluctuations are sufficient to explain some of these long-term step fluctuations, and a significant fraction of overall step variability.

These possibilities lead to two hypotheses. If walking steps could be decomposed into long- and short-term components, one hypothesis is that the short-term components, occurring over no more than a few steps, would be responsible for much of the variability in lateral foot placement or step width that has been observed previously [3]. The other hypothesis is that long-term variability could be due to slow, spontaneous fluctuations in walking speed. Speed fluctuations might explain much of the variability in fore-aft foot placement or step length, but not of step width, because the strongest known relationship is between step length and speed. If these possibilities are supported by experiment, then short-term variability could potentially highlight balance-related aspects of walking, and long-term fluctuations could quantify other, as yet unexplained, aspects of CNS variability.

The purpose of the present study was to test these hypotheses in the gait of healthy, young adults. Such a test requires quantification of both speed and step parameters, preferably over many steps of normal, over-ground walking. The steps should be sufficient in quantity and accuracy to estimate step variability. The walking speed should be relatively unconstrained, so that subjects can both select their preferred speed and allow it to fluctuate spontaneously. We therefore examined recordings of hundreds of unconstrained, over-ground steps to test these hypotheses. We tested whether step variability can be decomposed into two components, the first consisting of short-term variations in step width related to control of lateral balance, and the other of longer-term variations in step length due to fluctuations in walking speed. If these components are separable, they may prove useful for understanding the causes of gait variability.

## Methods

We examined step parameters from healthy adults walking over-ground at self-selected speed. Using a mobile motion capture system [3], we measured data including walking speed, step length, and step width, and their variabilities, from many contiguous steps per subject. These data were used to test for the presence of speed-related trends, such as the preferred step length relationship, that might explain a portion of the observed variabilities. Removing those trends, the remaining de-trended signals were tested for differences between step length and width and for an effect of walking with reduced visual feedback (eyes open vs. closed). We also tested whether the speed-related trends could be considered too slow to be related to balance, in terms of step-by-step variations.

### Experimental Methods

Data were collected in a previous study [3], briefly summarized here. Fourteen healthy young adult subjects (10 male, 4 female, 21-37 yrs., leg length 0.95 ± 0.06 m) walked over-ground at self-selected speeds. The experimental conditions tested were an eyes-open condition where subjects were asked to walk normally and at comfortable, self-selected speed toward a traffic cone, and an eyes-closed condition where subjects walked with their eyes closed and were asked to follow the sound of a portable radio carried about three meters ahead of them as a direction cue for relatively straight walking. They performed at least 4 independent trials per condition for about 100 consecutive steps each. To eliminate filtering artifacts from trial boundaries, each trial was trimmed by eight steps at each end. There were at least 80 or more contiguous steps remaining in each trial, as a larger number of steps aid estimation of step variability [24]. An additional fifteenth subject was recorded previously [3] but excluded from this study because of a lack of sufficient contiguous steps.

### Ethics Statement

Recruited subjects gave written informed consent according to Institutional Review Board procedures of the University of Michigan.

Step kinematics were recorded using a magnetics-based tracking system (MotionStar, Ascension Technology, Milton, VT), mounted on a portable cart that was rolled alongside subjects as they walked. Markers were placed on each forefoot and at the sacrum. Marker positions relative to the cart were collected at 100 Hz, and then filtered using a 3rd order Butterworth low-pass digital filter with a cut-off frequency of 6Hz, applied forward and backward in time. Marker positions relative to ground were obtained adding positions relative to the cart to an estimation of the cart’s motion relative to ground, similar to an algorithm used previously [3]. Step parameters were computed from ground-referenced marker positions following heel-strike, yielding lengths and widths for each step, treating the direction of travel as forward. Step period was also calculated, based on the time between consecutive steps. The sacral marker was used to estimate overall walking speed, which was also smoothed with a moving average filter with a window encompassing the samples of one preceding and one succeeding step. Thus, walking speed as defined here refers to the overall movement of the body, and is independent of the measurement of discrete steps, to reduce sensitivity to the corrective actions that might affect timing or distance of a single step. This measure of the body’s instantaneous speed is therefore separate from (but of course related to) each individual foot’s speed.

### Analysis

**Figure 1:**
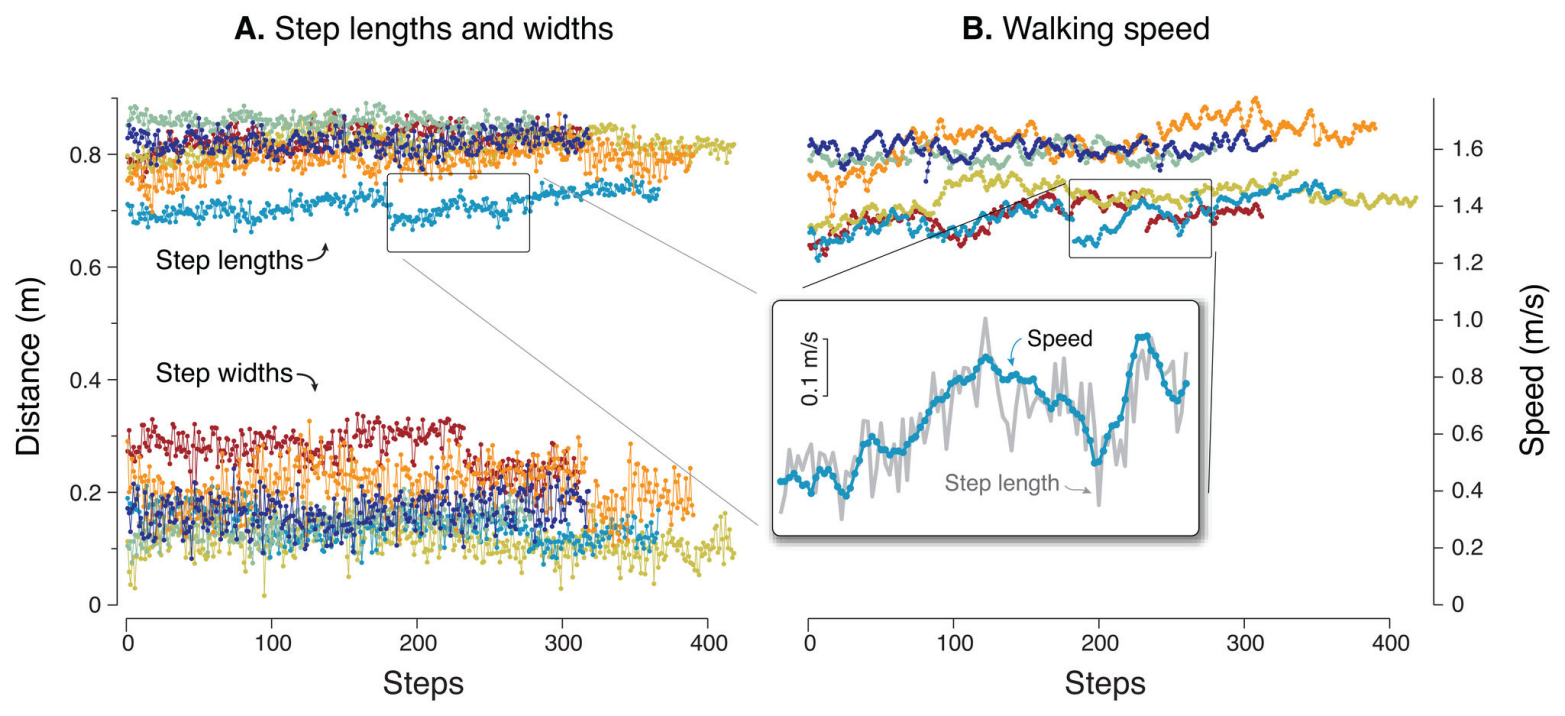
Step-by-step variability of overground walking. (**A**) Step length and width are observed to vary, apparently randomly, over many overground steps (different color for each subject). (**B**) Walking speed also fluctuates, albeit more slowly. Examination of speed and step length together (scaled and overlaid in inset diagram) reveals that the two may in fact co-vary. Data shown are all trials performed by six representative subjects.

We used foot kinematics to compute step length and width sequences for each trial (Figure 1). These were then examined for speed-related trends, which were used to decompose data into speed-related and speed-independent components, as well as low- and high-frequency components. We used step variance for the quantification of variability and for statistical comparisons. Variances of a signal’s separated components will sum linearly to equal the total variance, provided the components are uncorrelated. Although root-mean-square (or standard deviation, as recorded previously [3]) data were not used in comparative tests, such data are reported in the text as coefficient of variation (c. v.), defined as the standard deviation (s.d.) of step distances divided by their mean.

**Figure 2:**
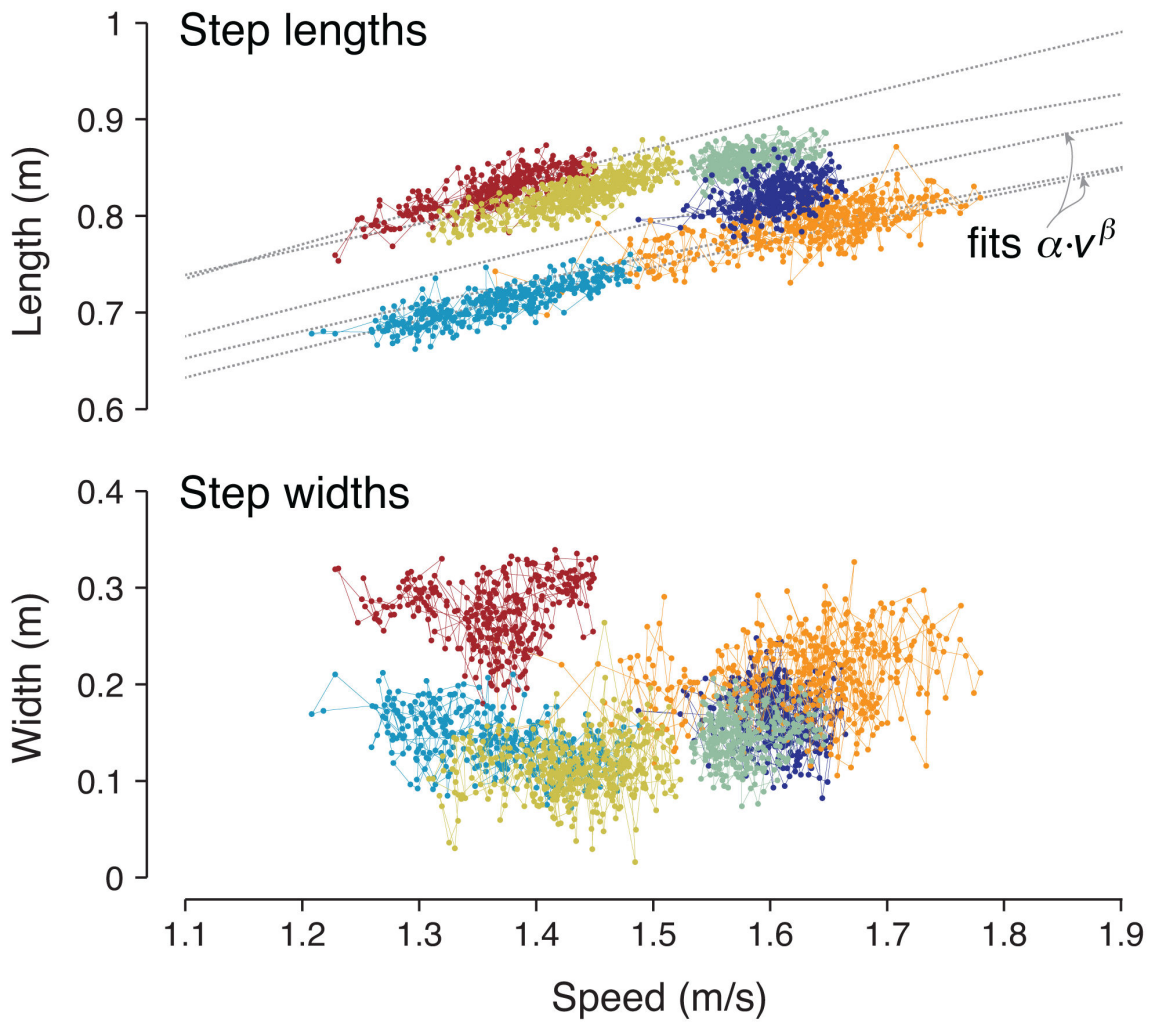
Representative step lengths and step widths as a function of walking speed. Step lengths are shown along with subject-specific curve fits (Eqn. 1) for the preferred step length vs. speed (*s* vs. *v*) relationship, *s* = α · *v* ^β^ [19], with typical values of β ranging 0.27–0.55. Step width exhibits little dependence on walking speed, and appears to have greater variability than step length. Data shown are trials from six subjects, with a different color for each subject.

We tested for speed-related trends within step lengths or widths (Figure 2). Mean step length *s* has been reported to vary with walking speed *v* through the equation [25,26]


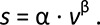
(1)

The logarithm of this equation yields log *s* = log α + β · log *v*, from which a linear regression yields estimates of empirical parameters α and β for each subject. Typical ranges reported [25] for α are 0.95–1.42, and for β are 0.27–0.55. For each subject, all trials in a single condition were analyzed together to yield a single combination of α and β. These parameters also implicitly determine step frequency, which is speed divided by step length. No speed-related trend has been reported for step width *w*, nor is such a trend expected here. To test for such a possibility we fitted a simple linear relationship with speed, *w* = *c* · *v* + *d*.

**Figure 3:**
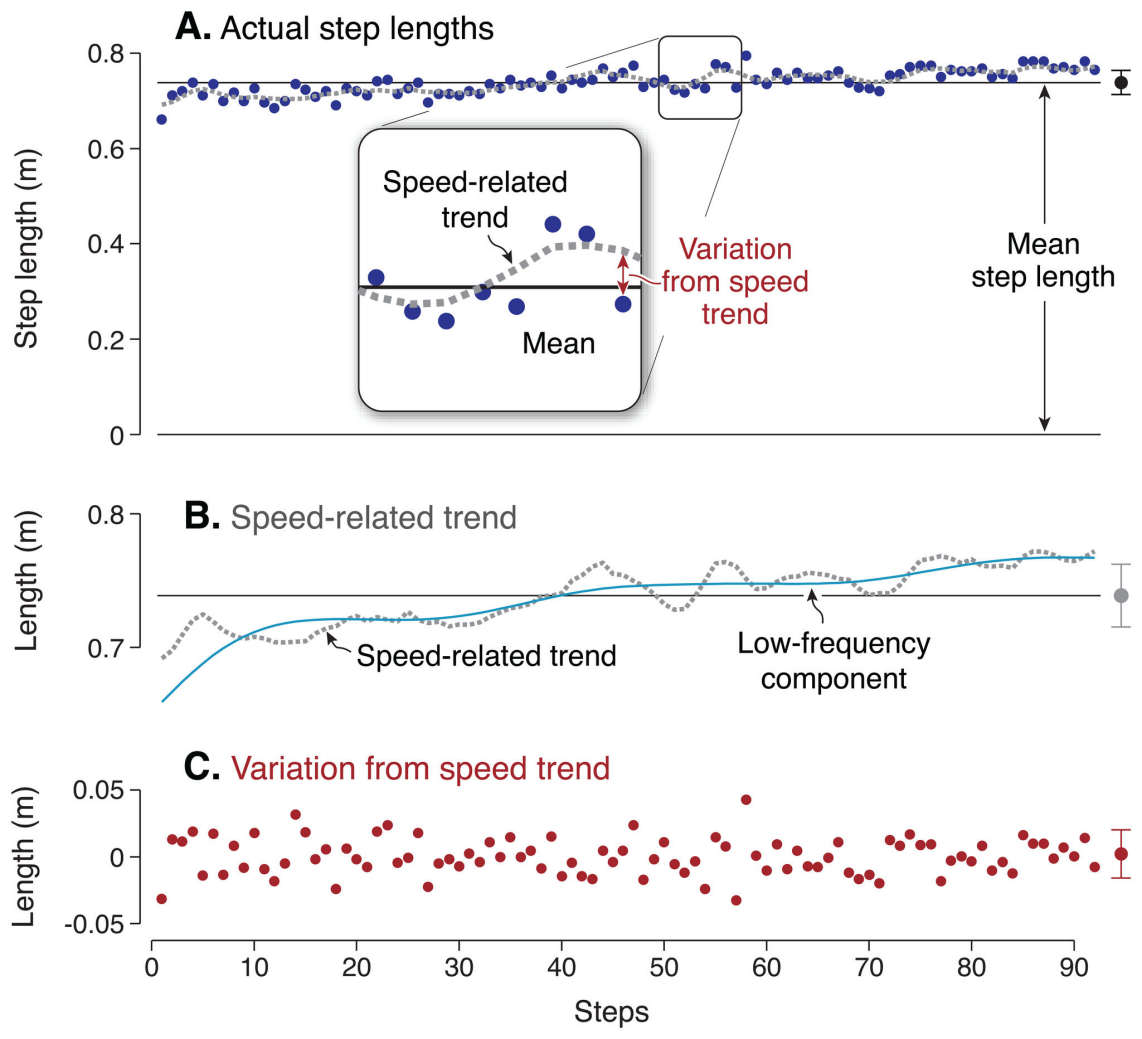
Decomposition of step lengths into a speed-related trend and variations from that trend. (**A**) Actual step lengths are hypothesized to vary according to the preferred step length vs. speed relationship, as walking speed fluctuates slowly during over-ground walking (see Eqn. 1). (**B**) The speed-related trend fluctuates in close correspondence to the low-frequency, long-term components of step length (solid line), found by low-pass filtering. (**C**) Variations from the speed trend, or de-trended step length, appear similar to random noise. This example shows one trial from a representative subject over about 90 steps. Variability of both the speed-related trend and de-trended step length are comparable in magnitude (mean and s.d. shown as error bars at right).

We next decomposed total step variability according to the identified speed-related trends (Figure 3). We subtracted the speed-related trend (e.g., Eqn 1) from the actual total step distance to yield the de-trended step length or width (Figure 3A). One portion of total step variability is therefore explained by fluctuations in walking speed, whereas the residual, de-trended variability is considered unexplained.

To examine the frequency content of step variability, we also decomposed step data into slower and faster components using a simple low-pass filter (Figure 3B). We propose that fast variations from one step to the next might be related to balance, whereas very slow variations should have little to do with balance, and might largely be explained by the speed-related trend (Eqn. 1). For this decomposition, we bi-directionally applied a third order Butterworth filter with a cut-off frequency of 0.033 steps^−1^ (period of 30 steps). We refer to the low-frequency filtered signal and its high-frequency residual as the long- and short-term components, respectively. We tested whether the de-trended step lengths were correlated with the long-term components, and similarly for step width. The cut-off frequency was chosen based on the assumption that step variations with periods of 30 steps or more are unlikely to be due to balance. We also tested for sensitivity to this parameter, and found cut-off periods of 10-30 steps to yield quite minor differences in numerical quantities (which are summarized in Table S4 of the Supporting Information), and to have no effects on any statistical conclusions reported here.

To characterize long-term correlations within the data, we computed the autocorrelations of speed, step length, and step width. Autocorrelation is the correlation between a signal and a time-lagged version of itself, computed as a function of the lag in steps. It has a value of unity for a time lag of 0, and will usually decrease with increasing lag for non-periodic noisy signals. White noise has no correlation with itself, and so the autocorrelation is zero for all lags but zero. A low frequency random process, generated by feeding white noise through a first-order low-pass filter, will have an exponentially decreasing autocorrelation, with a correlation time constant (in steps) characterizing how the exponential decays: A long time constant indicates a long-term trend. We used autocorrelation to qualitatively test for long-term correlations, and whether de-trending by speed also removes long-term correlations.

All measures were analyzed in dimensionless form. Displacement measures were normalized by each subject’s leg length, *L*, defined as the greater trochanter height. Speed measures were normalized by (*gL*)^0.5^, where *g* is gravitational acceleration. Time measures were normalized by *L* ^0.5^ *g* ^−0.5^. For reporting purposes, statistical data were converted back from dimensionless units to SI units using an average leg length of *L* = 0.954 m, an average speed normalization factor of (*gL*)^0.5^ = 3.04m·s^−1^, and an average time factor of *L* ^0.5^ *g* ^−0.5^ = 0.312 s.

We performed statistical tests to test for speed-related trends, and to test whether there were differences between step length and width variabilities. The indicator of a significant trend was the *P*-value of the regression coefficient (β for step length and *c* for step width). We used a two-way repeated measures ANOVA to test for a significant effect of length vs. width, and eyes open vs. closed as the factors, followed by post hoc t-tests for each factor, with the Holm-Sidak step-down criterion for multiple comparisons [27]. The threshold for significance was selected as *P* < 0.05. All trials were separately analyzed in terms of left-to-right steps and right-to-left steps, to yield summary measures such as a mean left–right step length and a mean right-to-left step length. These measures were then averaged together to yield a single summary measure (e.g., average step length or step width) for each trial.

## Results

All subjects walked at fairly steady speed, but with slow, continuous fluctuations throughout each trial. The walking speed in normal eyes-open conditions was an average of 1.51 ± 0.08 m·s^−1^ (mean ± s.d.), with a step length of 0.792 ± 0.036 m and step width of 0.168 ± 0.044 m. The coefficients of variation were 2.3% for speed, 2.0% for step length, and 15.4% for step width. As detailed below, step lengths were found to follow the preferred relationship with speed, even though the spontaneous fluctuations in speed were quite small. In contrast, step width did not follow a significant trend with speed, and did not exhibit long-term correlations. Walking with eyes closed had little substantive effect except an increase in variability (two-way ANOVA, *P* < 5·10^−4^ for both eyes open and width/length factors, interaction *P* = 0.0086), particularly for short-term step width. We first summarize results for walking with eyes open, and then examine the differences that occurred with eyes closed. Unless otherwise noted, statistical tests and *P*-values reported below refer either to a linear regression (referred to as significant trend or correlation), or to a post-hoc paired t-test comparing two conditions or measures (referred to as significant difference). Major results are also tabulated in the Supporting Information, including average step parameters (Table S1), step variabilities expressed as variance (Table S2), and step variabilities expressed as root-mean-square values (Table S3).

### Effects of walking speed

**Figure 4:**
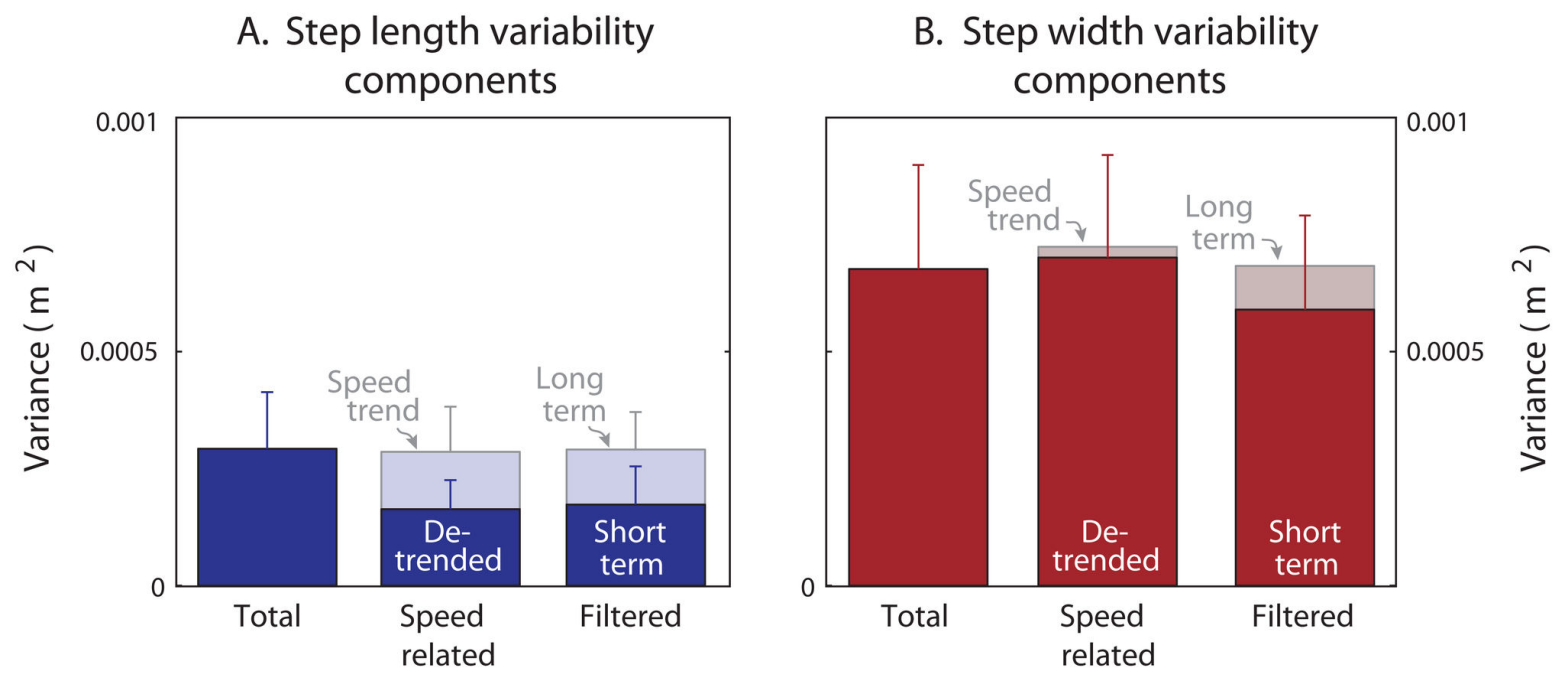
Overall variance of step length and step width. (**A**) Step length and (**B**) step width are each decomposed by de-trending and by filtering (*N* = 14). In each panel, the average total variability (labeled “Total” at left, with error bars for standard deviation across subjects) is contrasted with the variability of the components determined by the “Speed-related” trend and the “Filtered” components (middle and right of each panel). The speed-related trend and the de-trended step lengths (lighter and darker bars respectively) have similar variances whose sum is nearly equal to total variance. The long- and short-term components (lighter and darker bars respectively) of step length, separated by filtering, yield variances comparable to the speed-related components. In contrast, step width exhibits little speed-related trend, and little low-frequency content. The speed-related trend accounted for about half of the total variance in step length, but a negligible amount of width. De-trended step width variability was over 4 times the de-trended step length variability (*P* = 4.2 · 10^−8^).

For walking with eyes open, step length exhibited a strong dependence on walking speed (Figure 2). Fitting step length *s* to speed *v* from all of each subject’s trials according to the preferred relationship *s* = α · *v* ^β^ (Eqn. 1) yielded a significant correlation (*P* = 1.4 · 10^−16^) with R^2^ = 0.59 ± 0.12. The corresponding coefficients α = 1.22 ± 0.11 (mean ± s.d.) and exponent β = 0.54 ± 0.10, were comparable to the range reported by Grieve [19]. In contrast, step width was not significantly correlated with speed (R^2^ = 0.063 ± 0.12, *P* = 0.079), with linear coefficient *c* = 0.166 ± 0.368 and offset d = 0.091 ± 0.186. Within individual trials as well, walking speed accounted for a substantial amount of step length variability, but not step width variability (Figure 4). Using walking speed and each subject’s overall preferred relationship to de-trend their steps, the speed-related trend accounted for 42% of the total variance in step length on a trial-by-trial basis. (This is lower than the R^2^ = 59% across all of each subject’s trials, because there was less speed variation within a single trial and thus a smaller range of step lengths accounted for.) But for step width, walking speed only accounted for 3.4% of the variance per trial.

Removal of the speed-related trends was found to accentuate the raw differences between step width and step length variability (Figure 4). During normal eyes-open walking, de-trended step width variance was 4.3 times the step length variance (*P* = 4.2 · 10^−8^).

The effect of removing speed-related trends was similar to the effect of filtering the same signals. The long-term components (correlations over 30 steps or more) of step length accounted for 40% of the variance, whereas the long-term components of step width accounted for only 14% of the variance (compare Figure 4A and Figure 4B). Moreover, the speed-related trend in step length was strongly correlated with long-term step length, with R^2^ = 0.70 (*P* = 0.035). There was no significant correlation between the speed-related trend and the long-term component of step width, with R^2^ = 0.02 (*P* = 0.17). Both methods of decomposition yielded variances that summed to nearly equal the total variance, indicating negligible linear dependence between complementary components.

### Effects of reduced vision

**Figure 5:**
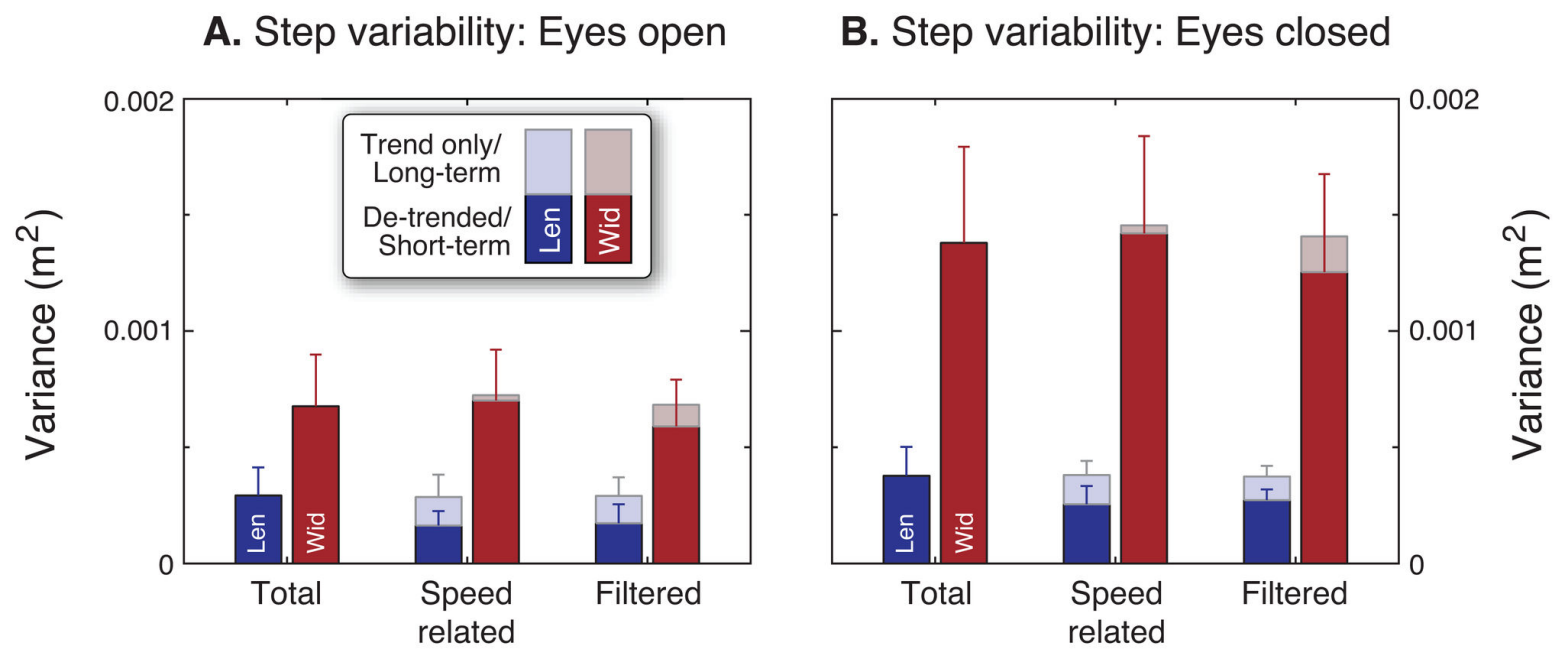
Difference in step length and width variability between walking with eyes open and eyes closed. (**A**) Eyes open. (**B**) Eyes closed. Each panel shows total variance of step length and width (left and right bars of each pair), speed-related length and width components, and filtered length and width components. When walking with eyes closed, subjects walked with 56% greater step length variance, and 103% greater step width variance (*P* < 0.05). Speed-related trends do not account for the increased gait variability with eyes closed.

Walking with eyes closed had relatively little effect on average steps, and much greater effect on step variability (Figure 5). Subjects walked with slightly reduced average step lengths and speeds, with reductions of 3.8% and 4.5%, respectively (*P* < 0.05). But both eyes open and closed trials were consistent with the same preferred step length relationship, that is, with no significant differences in α and β (*P* = 0.36 and *P* = 0.17, respectively). Mean step width exhibited a non-significant increase of 6.4% (*P* = 0.09). The greatest effect of the eyes closed condition was on the step variabilities with the speed-related trends excluded. There was a 103% increase in de-trended step width variability (*P* = 5.7 · 10^−6^), and about half that increase, 56% in de-trended step length variability (*P* = 0.001).

**Figure 6:**
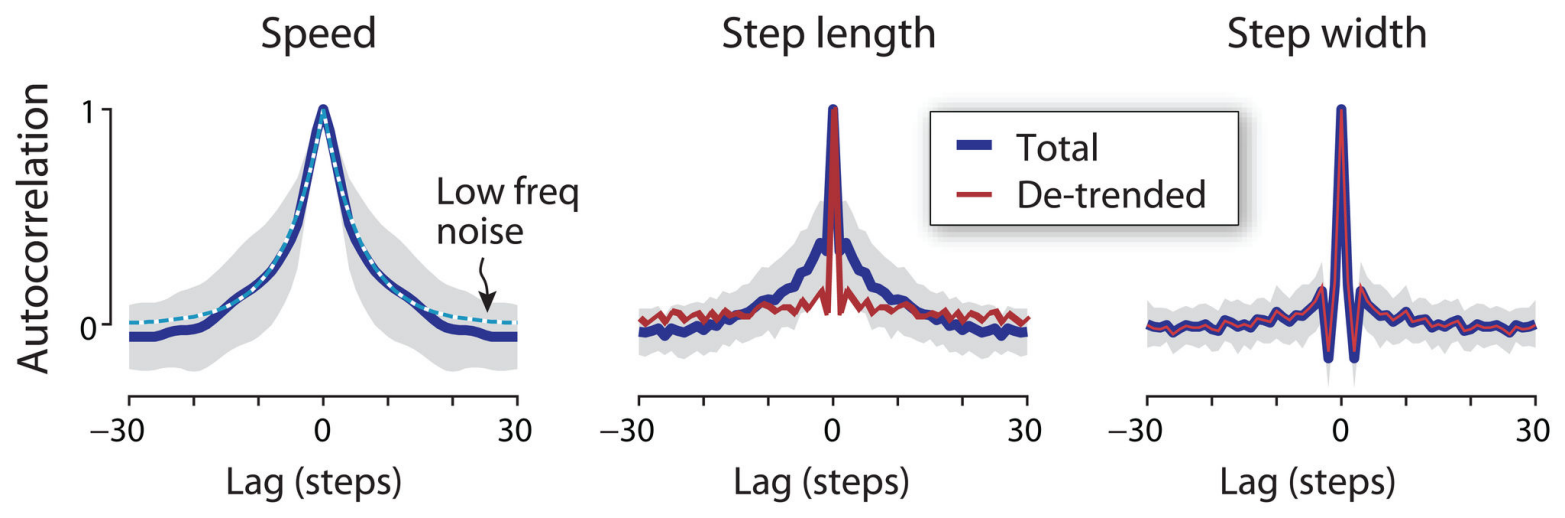
Autocorrelation of walking speed, step length and step width as a function of lag in steps. Autocorrelation of speed decreases gradually with lag (solid line denotes mean, shaded region denotes ± 1 s.d.), comparable to the long-term correlations of low-frequency noise (dashed line; 6 step correlation time constant). Total step length autocorrelation also exhibits a long-term trend, whereas de-trended step length has an autocorrelation resembling white noise. Step width autocorrelation exhibits very little long-term correlation, and de-trending has little effect on the autocorrelation. (White noise has an autocorrelation of 1 at zero lag, and 0 at all other lags.).

De-trending was found to affect the autocorrelation of step length much more so than step width (Figure 6). Walking speed exhibited an exponentially decreasing autocorrelation, resembling a low frequency noise process with a correlation time constant of 6 steps. Total step length also bore a resemblance to low-frequency noise, but removal of the speed-related trend resulted in a de-trended step length that resembled white noise. In contrast, total step width did not exhibit an exponentially decreasing autocorrelation, but instead showed a negative correlation at a lag of one step, and a positive correlation at a lag of two steps. In other words, if one step were wider than average, it was usually followed by a slightly narrower step on average, and then by a very slightly wider step on average. Moreover, step width was virtually unaffected by de-trending. Step width varied substantially from step to step with short-term correlations dissimilar to white noise, and this structure was quite distinct from the long-term trends found in step length.

## Discussion

We had sought to determine whether step variability is associated with the spontaneous speed fluctuations that occur during steady walking, and whether such an effect is separate from the control of balance during walking. Our data revealed slow, spontaneous fluctuations in walking speed, which accounted for much of the variability observed in step length. They also revealed practically no such trend in step width, which varied only with shorter-term fluctuations. While previous data have shown greater variability in step width than step length [3,13], that observation is accentuated by accounting for the speed-related trend. After removing the effects of walking speed, there was four times as much step width variability as step length variability, a difference further magnified during walking with eyes closed. We interpret these findings to mean that step variability may have independent components separable in both direction and time. One component is relatively slow and largely in the fore-aft direction, associated with slow fluctuations in walking speed. Another component is much faster and in the lateral direction, associated with step-to-step control of balance. These independent components may provide insight regarding the control of walking.

Our first observation is that self-selected speed fluctuates during over-ground walking (Figure 2). The fluctuations were about 2.3% (c.v.) and resemble low frequency noise, as from white noise passed through a low frequency process filter (Figure 6). Their relatively long correlation time constant (about 6 steps) is considerably greater than what would be expected from the dynamics of walking [12,28], unless explicitly perturbed by low-frequency noise. The fluctuations were also considerably slower for speed than for step length and width, which both exhibited much faster correlation time constants. Over short time scales, it is expected that speed should be correlated between successive steps (lags near zero steps in Figure 6), in part due to step-by-step adjustments to step length. But there were also long-term correlations in speed and step length that appear too slow to be related to balance.

The slow fluctuations in speed explained about half of total step length variability (Figure 4). Subtracting the trend due to the step length vs. speed relationship, the remaining unexplained step length variability was quite small, about 1.5% (c. v.) of the preferred step length, and exhibited much less long-term content (Figure 6). The remaining fluctuations occurred more on a step-to-step basis, quite similar to applying a high-pass filter to the data (Figures 3 and 5). The variability increased slightly with eyes closed (Figure 5A vs. B), suggesting that a fraction of the de-trended variability may indeed be associated with balance control. But the most readily identified contribution to overall step length variability is associated with slow fluctuations in speed.

This contrasted with step width variability, which exhibited neither a speed-related trend nor substantial low-frequency content. Each step’s width was most correlated with the neighboring one or two steps in time, indicative of a process with short-term dynamics (and short correlation time constants, Figure 6). Step width variability has been proposed to result in part from active, integrative sensorimotor control of lateral balance [7,8]. This was suggested by a dynamic walking model with passive instability in the lateral direction but not fore-aft [12], as well as empirical observations [3]. Step width variability also appears to be especially sensitive to both visual [10] and physical [14,29] perturbations. If lateral foot placement was not controlled and simply allowed to vary randomly, we would not expect it to be sensitive to visual feedback. But lateral perturbations to visual feedback cause substantial increases in lateral step variability, whereas fore-aft perturbations have little effect on fore-aft variability [10]. In addition, removal of vision causes a greater increase in lateral step variability than fore-aft [3]. This suggests that lateral foot placement may indeed be actively controlled, in part by vision. The present results reinforce those observations, with de-trended step width variability approximately doubling in the eyes closed condition. De-trended (i.e. short-term) step length variability also increased, but by about half the proportion. Removal of the speed-related trend makes the greater sensitivity of step width even more apparent than seen previously. Without visual information, one may have less precise sense of the body’s general balance [10,30]. With poorer balance sense, the determination of where the foot should be placed may be less certain. Greater step width variability may then be indicative of greater lateral unsteadiness [7,8]. The effect of closing the eyes on step width variability but not step length variability could mean that lateral foot placement is governed in part by vision, and perhaps for maintenance of balance.

As for the slow fluctuations, their origin is unclear. They could be produced by a variety of low-frequency perturbations, for example to the torques produced by the joints. Whether such perturbations affect one variable or another, the preferred step length relationship appears to be quite well enforced, so that both step length and speed have well-correlated slow components. In accounting for half of the total variance in fore-aft foot placement, walking speed actually has considerably more unexplained variability than step length or timing. This suggests the possibility that the fluctuation is in the self-selected set-point for speed. In the absence of an external speed cue from a treadmill, self-selection is at the whim of an individual subject, and that whim need not be constant even if it is in part under executive control. The set point could depend on a variety of complex factors (e.g., [31]), such as energy expenditure, the conscious desire to reach a destination within a given time, or degree of confidence or wakefulness. Such factors could vary with time, making the speed set-point a slowly time-varying quantity. The result would be slow, apparently random fluctuations observable within relatively long walking trials, or even as inter-trial variability between shorter trials. While slow fluctuations remain unexplained, they appear to contribute to variability, particularly in speed and step length.

Even with fluctuations in speed, subjects adhere quite closely to the preferred step length relationship. That behavior is energetically favorable because the preferred step length corresponds with a minimum of metabolic energy expenditure for a given speed [16]. Step length appears to be determined in large part by the amount of push-off provided by the leg in late stance [32], and the low variability observed implies that push-off is regulated with relatively high precision as well. Low variability may also be favored by the coupled dynamics of the limbs and local reflexes, which may have self-stabilizing tendencies that automatically counter perturbations [29]. Those dynamics are such that a slightly longer (or shorter) step results in a heel-strike collision that dissipates relatively more (or less) energy, so that the following step automatically tends toward the mean [31]. Little integrative feedback of sensory information is therefore needed to counter small perturbations. This does not, however, explain how the preferred step length is regulated so precisely, but other studies indicate that there is quite a strong and fast attraction toward the preferred step length relationship that does use integrative feedback [33].

Measurements of step variability are often employed in assessment of sensorimotor function, for example in older adults [1,5,34]. Any variability estimate is limited by the precision of the measurement apparatus. Measurement noise acts as a lower bound or noise floor that adds to actual step variability to yield the (imperfectly) observed step variability. If observed step variability is to be used for functional assessment of gait, it is therefore best for the actual variability to be high relative to the noise floor, and also exhibit high sensitivity to the conditions that affect gait. Step length has relatively low unexplained variability and little sensory dependence, perhaps making its variability a poor indicator of sensorimotor integration and control. In contrast, step width has much greater variability (e.g. more than four times the variance than step length, and 15.4% c. v. compared to 2.3% or less for step timing, length, and speed). Not only is step width variability highly dependent on integrative sensing [10], but it has also been shown to increase with age [13] and with sensory deficits [30]. The main drawback is that step width variability is difficult to measure accurately for many over-ground steps. Fortunately, it does appear to exhibit similar sensitivities during treadmill walking [13], for greater ease of measurement. Whether measured over-ground or on a treadmill, we expect step width variability to be a practical and sensitive measure of sensorimotor control for balance for relatively normal gait.

Other step variability measures may quantify other forms of gait function less directly related to balance. We detected fluctuations in walking speed, step length, and step timing that appear to be too slow to be associated with balance, but may yet be useful indicators of other factors such as executive function and physical motor capacity. In particular, step timing variability is easy to measure and exhibits a form of long-term trend (e.g., de-trended fluctuation analysis [35]) that is correlated with gait pathologies. It is unclear what causes these trends, but they might be associated with slow, spontaneous fluctuations in walking speed. This is consistent with observations that the long-term trends behave differently on a treadmill, where walking speed fluctuates little and body position must be regulated relative to the treadmill belt [21,33]. (See Figure S1 in the Supporting Information for a consideration of the effects of treadmill walking on the analysis presented here.) Whatever their cause, long-term trends may still be indirectly related to balance, through for example subjective confidence. We found long-term fluctuations to be easily isolated with low-pass filtering, yielding variabilities that may serve as indicators of gait function complementary to, rather than redundant to, step width variability.

There are a number of limitations to this study. We attempted to measure relatively unconstrained walking. It is possible that the contrivance of “walking normally” with motion capture may have affected self-selection of walking speed. There are also limitations to accurate measurement of steps and step variability over long distances, which would have affected results. Assuming the observed speed-related trends apply to normal walking, there also remains the question of what explains the remaining variability. The present results cannot explain why walking speed is not constant, and what other factors affect step parameters, nor can they prove the relationship between step variations and balance. In addition, our hypotheses are based on a dynamic walking model [12], which is particularly unstable in the lateral direction without active foot placement. It also exhibits negligible coupling between long-term components of speed and step length. Some of these behaviors might result from the many simplifications of the model, and not capture the complexities of actual human walking. It would therefore be helpful to consider alternative models of walking stability.

We have identified two independent contributions to step variability. We found that short-term variations in foot placement occur mostly laterally. Step width variability appears to be a robust indicator of step-to-step balance during walking, especially when measured accurately over a sufficient number of steps. We also found slow fluctuations in walking speed that determine about half of the observed long-term fluctuations in step length. This is because young, healthy adults follow the preferred relationship between step length and speed to a precision greater than the maintenance of speed. There are almost certainly other contributions to step variability, but the identification of these two components suggests that variability measurements may prove useful not only as indicators of poor function, but also as probes of the fundamental mechanisms underlying gait.

## Supporting Information

Table S1 Average step parameters. (N = 14, mean ± s.d.). Significant difference between conditions is indicated by asterisk (*, P < 0.05).
(PDF)

Table S2 Step variabilities, expressed as variance. (N = 14, mean ± s.d.). Normalization units are given in terms of leg length L and gravitational acceleration g. Short-term variability is defined by applying a high-pass filter to step data, with a cut-off period of 30 steps (and long-term by a low-pass filter).
(PDF)

Table S3 Step variabilities, expressed as root-mean-square values. (N = 14, mean ± s.d.). Units are in SI, using the mean normalization factors to re-dimensionalize the data.
(PDF)

Table S4 Results from parameter study on the filter to separate short- and long-term components. Step variabilities of Table S2 (variance) and Table S3 (RMS variability) are recomputed here with filter cut-off period of 10 steps (rather than 30 steps). The choice of filter causes minor differences in tabulated results, but statistically significant findings remain unchanged: The correlation between speed-related and long-term step lengths remained significant with R2 = 0.78 (P = 0.0005), and the correlation for step widths remained insignificant with R2 = 0.03 (P = 0.14).
(PDF)

Figure S1 Sample stride length data for one foot of a young adult walking over-ground compared to the same person walking on a treadmill. Contiguous strides are shown, along with error bars indicating mean and standard devations. Walking speed fluctuates somewhat during over-ground walking, contributing some variability to stride length due to the stride length vs. speed relationship alone. Treadmill walking places an additional constraint on speed, leading to a smaller contribution of the stride length vs. speed relationship to stride length variability. For the trials shown here, speed variance was 96.2% greater over-ground than on treadmill, and stride length variance was 88% greater (and in terms of RMS variability, 40.1% more and 37.2% more, respectively).
(PDF)
