# Supplementary material for: Two Independent Contributions to Step Variability during Over-Ground Human Walking
Source: PLoS One. 2013 Aug 28;8(8):e73597. doi: 10.1371/journal.pone.0073597 (PMC3756042; doi:10.1371/journal.pone.0073597)
Supplement: Table S1 — (N = 14, mean ± s.d.). Significant difference between conditions is indicated by asterisk (*, P < 0.05). (PDF) [file pone.0073597.s001.pdf]

Supporting Information

Two independent contributions to step variability during over-ground human walking

S. H. Collins and A. D. Kuo

Table S1 Average step parameters (N = 14, mean ± s.d.). Significant difference between conditions is indicated by asterisk (*, $P < 0.05$).

Average	units	Eyes Open condition	Eyes Closed condition
Speed	$m \cdot s^{-1}$	1.510 ± 0.084	1.442 ± 0.104*
Step length	m	0.792 ± 0.036	0.762 ± 0.055*
Step width	m	0.168 ± 0.044	0.179 ± 0.047
Step frequency	s	1.814 ± 0.113	1.806 ± 0.119
