# Supplementary material for: Two Independent Contributions to Step Variability during Over-Ground Human Walking
Source: PLoS One. 2013 Aug 28;8(8):e73597. doi: 10.1371/journal.pone.0073597 (PMC3756042; doi:10.1371/journal.pone.0073597)
Supplement: Table S2 — (N = 14, mean ± s.d.). Normalization units are given in terms of leg length L and gravitational acceleration g. Short-term variability is defined by applying a high-pass filter to step data, with a cut-off period of 30 steps (and long-term by a low-pass filter). (PDF) [file pone.0073597.s002.pdf]

Supporting Information

Two independent contributions to step variability during over-ground human walking

S. H. Collins and A. D. Kuo

Table S2 Step variabilities, expressed as variance ($N = 14$, mean ± s.d.). Normalization units are given in terms of leg length L and gravitational acceleration g. Short-term variability is defined by applying a high-pass filter to step data, with a cut-off period of 30 steps (and long-term by a low-pass filter).

Variance	units	Eyes Open condition	Eyes Closed condition
Totals			
Speed	gL	0.000133 ± 0.000065	0.000122 ± 0.000058
Step length	L^2	0.000292 ± 0.000121	0.000377 ± 0.000125
Step width	L^2	0.000677 ± 0.000221	0.001380 ± 0.000413
De-trended			
Step length	L^2	0.000163 ± 0.000061	0.000255 ± 0.000079
Step width	L^2	0.000700 ± 0.000219	0.001420 ± 0.000418
Speed trend			
Step length	L^2	0.000121 ± 0.000097	0.000125 ± 0.000061
Step width	L^2	0.000023 ± 0.000028	0.000034 ± 0.000048
Short-term			
Step length	L^2	0.000173 ± 0.000069	0.000272 ± 0.000090
Step width	L^2	0.000590 ± 0.000201	0.001254 ± 0.000421
Long-term			
Step length	L^2	0.000117 ± 0.000081	0.000101 ± 0.000046
Step width	L^2	0.000093 ± 0.000044	0.000154 ± 0.000061
