# Supplementary material for: Two Independent Contributions to Step Variability during Over-Ground Human Walking
Source: PLoS One. 2013 Aug 28;8(8):e73597. doi: 10.1371/journal.pone.0073597 (PMC3756042; doi:10.1371/journal.pone.0073597)
Supplement: Table S3 — (N = 14, mean ± s.d.). Units are in SI, using the mean normalization factors to re-dimensionalize the data. (PDF) [file pone.0073597.s003.pdf]

Supporting Information

Two independent contributions to step variability during over-ground human walking

S. H. Collins and A. D. Kuo

Table S3 Step variabilities, expressed as root-mean-square values (N = 14, mean ± s.d.). Units are in SI, using the mean normalization factors to re-dimensionalize the data.

RMS Variability	units	Eyes Open condition	Eyes Closed condition
Totals			
Speed	$m \cdot s^{-1}$	0.0343 ± 0.0088	0.0330 ± 0.0073
Step length	m	0.0160 ± 0.0034	0.0183 ± 0.0029
Step width	m	0.0245 ± 0.0039	0.0351 ± 0.0053
De-trended			
Step length	m	0.0120 ± 0.0022	0.0151 ± 0.0023
Step width	m	0.0250 ± 0.0038	0.0356 ± 0.0053
Speed trend			
Step length	m	0.0099 ± 0.0037	0.0103 ± 0.0027
Step width	m	0.0038 ± 0.0026	0.0044 ± 0.0035
Short-term			
Step length	m	0.0123 ± 0.0024	0.0156 ± 0.0024
Step width	m	0.0229 ± 0.0038	0.0333 ± 0.0056
Long-term			
Step length	m	0.0098 ± 0.0034	0.0093 ± 0.0023
Step width	m	0.0090 ± 0.0021	0.0116 ± 0.0024
