# Supplementary material for: Two Independent Contributions to Step Variability during Over-Ground Human Walking
Source: PLoS One. 2013 Aug 28;8(8):e73597. doi: 10.1371/journal.pone.0073597 (PMC3756042; doi:10.1371/journal.pone.0073597)
Supplement: Table S4 — Step variabilities of Table S2 (variance) and Table S3 (RMS variability) are recomputed here with filter cut-off period of 10 steps (rather than 30 steps). The choice of filter causes minor differences in tabulated results, but statistically significant findings remain unchanged: The correlation between speed-related and long-term step lengths remained significant with R2 = 0.78 (P = 0.0005), and the correlation for step widths remained insignificant with R2 = 0.03 (P = 0.14). (PDF) [file pone.0073597.s004.pdf]

Supporting Information

Two independent contributions to step variability during over-ground human walking

S. H. Collins and A. D. Kuo

Table S4 Results from parameter study on the filter to separate short- and long-term components. Step variabilities of Table S2 (variance) and Table S3 (RMS variability) are recomputed here with filter cut-off period of 10 steps (rather than 30 steps). The choice of filter causes minor differences in tabulated results, but statistically significant findings remain unchanged: The correlation between speed-related and long-term step lengths remained significant with $R^2 = 0.78$ ($P = 0.0005$), and the correlation for step widths remained insignificant with $R^2 = 0.03$ ($P = 0.14$)

Parameter (10-step cut-off)	units	Eyes Open condition	Eyes Closed condition
Variance			
Short-term			
Step length	L^2	0.000120 ± 0.000047	0.000202 ± 0.000070
Step width	L^2	0.000483 ± 0.000172	0.001063 ± 0.000380
Long-term			
Step length	L^2	0.000159 ± 0.000097	0.000156 ± 0.000058
Step width	L^2	0.000163 ± 0.000055	0.000262 ± 0.000097

RMS Variability			
Short-term			
Step length	m	0.0103 ± 0.0019	0.0134 ± 0.0022
Step width	m	0.0207 ± 0.0036	0.0307 ± 0.0054
Long-term			
Step length	m	0.0116 ± 0.0035	0.0117 ± 0.0022
Step width	m	0.0120 ± 0.0020	0.0152 ± 0.0029
