# Supplementary material for: Two Independent Contributions to Step Variability during Over-Ground Human Walking
Source: PLoS One. 2013 Aug 28;8(8):e73597. doi: 10.1371/journal.pone.0073597 (PMC3756042; doi:10.1371/journal.pone.0073597)
Supplement: Figure S1 — Contiguous strides are shown, along with error bars indicating mean and standard devations. Walking speed fluctuates somewhat during over-ground walking, contributing some variability to stride length due to the stride length vs. speed relationship alone. Treadmill walking places an additional constraint on speed, leading to a smaller contribution of the stride length vs. speed relationship to stride length variability. For the trials shown here, speed variance was 96.2% greater over-ground than on treadmill, and stride length variance was 88% greater (and in terms of RMS variability, 40.1% more and 37.2% more, respectively). (PDF) [file pone.0073597.s005.pdf]

Supporting Information

Two independent contributions to step variability during over-ground human walking

S. H. Collins and A. D. Kuo

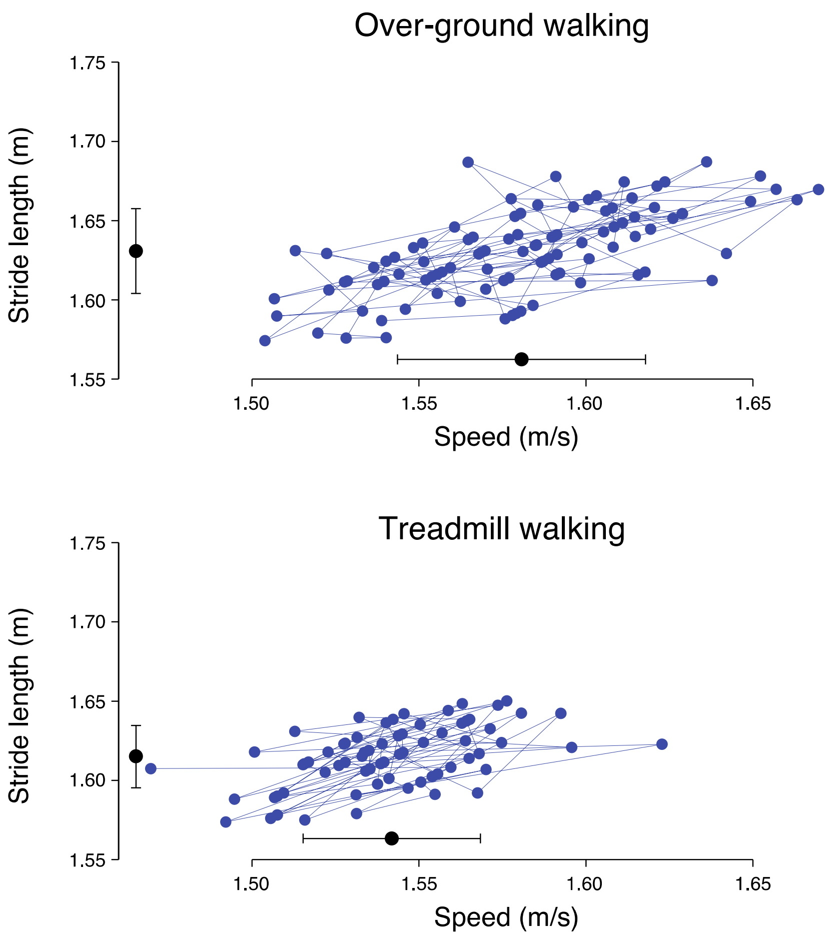

Figure S1 Sample stride length data for one foot of a young adult walking over-ground compared to the same person walking on a treadmill. Contiguous strides are shown, along with error bars indicating mean and standard devations. Walking speed fluctuates somewhat during over-ground walking, contributing some variability to stride length due to the stride length vs. speed relationship alone. Treadmill walking places an additional constraint on speed, leading to a smaller contribution of the stride length vs. speed relationship to stride length variability. For the trials shown here, speed variance was 96.2% greater over-ground than on treadmill, and stride length variance was 88% greater (and in terms of RMS variability, 40.1% more and 37.2% more, respectively).
